# Interspecific and intraspecific analysis of *Selinum* spp. collected from Indian Himalayas using DNA barcoding

**DOI:** 10.1186/s43141-022-00345-0

**Published:** 2022-04-22

**Authors:** Ravi Prakash Srivastava, Gauri Saxena, Lav Singh, Arpit Singh, Praveen C. Verma, Gurminder Kaur

**Affiliations:** 1 Department of Botany, University of Lucknow, Lucknow, Uttar Pradesh 226007 India; 2 Plant Molecular Biology & Genetic Engineering Laboratory, Council of Scientific and Industrial Research, National Botanical Research Institute (CSIR-NBRI), Lucknow, Uttar Pradesh 226001 India; 3 Institute of Bioscience and Technology, Shri Ramswaroop Memorial University, Deva Road, Barabanki, Lucknow, Uttar Pradesh 225003 India

**Keywords:** DNA barcoding, Medicinal plants, *matK*, *rbcL*, *Selinum*, Phylogenetic studies

## Abstract

**Background:**

DNA barcoding is a powerful method for phylogenetic mapping and species identification. However, recent research has come to a consistent conclusion about the universality of DNA barcoding. We used *matK* and *rbcL* markers to test the universality of twelve accessions from different locations belonging to two *Selinum* species, *Selinum tenuifolium* Wall. C. B. Clarke and *Selinum vaginatum* C. B. Clarke, keeping in mind their ability to identify species and establish phylogenetic relationships within and between the accessions.

**Results:**

The success rates of PCR amplification using *matK* and *rbcL* were 75.26% ± 3.65% and 57.24% ± 4.42%, and the rate of DNA sequencing was 63.84% ± 4.32% and 50.82% ± 4.36%, respectively, suggesting that success rates of species identification of the two fragments were higher than 41.00% (*matK*, 41.50% ± 2.81%; *rbcL*, 42.88% ± 2.59%), proving that these fragments might be used to identify species. The best evolutionary tree with good supporting values was produced utilizing combinations of *matK + rbcL* markers when phylogenetic relationships were built with random fragment combinations. The twelve accessions of *Selinum* collected from different locations and their molecular sequences of *matK* and *rbcL* markers were blasted with other genera of Apiaceae family, and it was found that *Selinum* is most closely related to *Angelica* species of Apiaceae family.

**Conclusion:**

The present study has grouped twelve accessions of *Selinum* species using molecular markers into phylogenies, which is first-of-its-kind report that established interrelationships within different species of Apiaceae with respect to *Selinum*.

**Graphical abstract:**

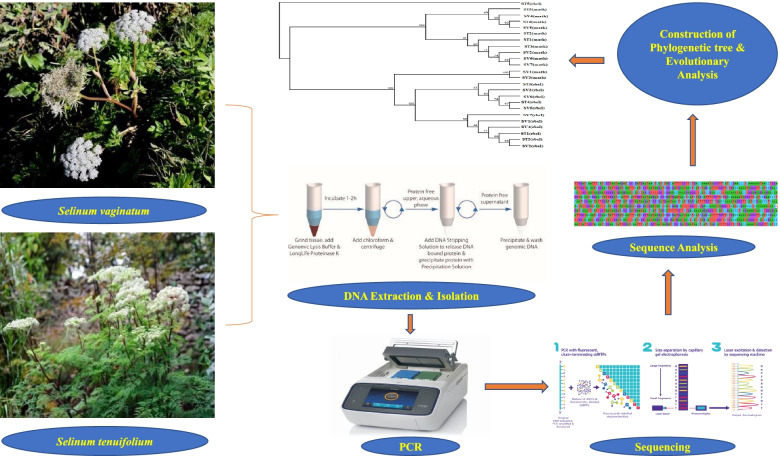

## Background

Since thousands of years, medicinal plants have been used in health-care systems [1, 2]. The correct identification of these plants is required for their safe use. Medicinal plants were traditionally classified by experienced experts based on morphological characteristics. However, it may take an expert many years to accurately identify these plants, and only a few taxonomists have been able to do so for around 1000 species so far [3]. Furthermore, the number of trained experts has been decreasing as the workload on identification has increased. Simultaneously, the number of adulterants and substitutes on the market is increasing, resulting in variation in the quality of medicinal plants [4, 5]. Thus, identifying medicinal plants is a significant challenge, posing safety risks to both the traditional medicine industry and consumers. As a result, a quick and accurate method for authenticating these plant species is required. DNA barcoding is a novel method for species identification that has the capacity to overcome the aforementioned challenges [3].. This method is based on sequence differences within short, standardized DNA regions, with the primary goal of both authenticating and discovering new species [3, 6, 7]. DNA barcoding is an efficient and low-cost technique that could boost the work of specialists while also making species validation accessible to nonspecialists [8–11]. Furthermore, it is independent of both morphological characteristics and expert experiences, allowing for faster, more subjective, and accurate results. As a result, DNA barcoding has quickly become the focus of research, and it has been recognized as a powerful tool for species identification and the construction of phylogenetic trees [12, 13].

The Apiaceae family contains approximately 250–440 genera and a range of 3300–3700 species that are widely distributed throughout the temperate zones of both hemispheres. *Selinum* belongs to Apiaceae is perennial, aromatic, and medicinal plant ranging from 1 to 2 m in length [14]. *Selinum* species are mainly found in Western Himalayas and Himalayan foothills of India, West Pakistan, Bhutan, China, and Nepal. Some of its species have also been reported from Europe, specifically in the UK, Poland, Bosnia, and Croatia. Ecological and floral diversity type studies so far do not present the molecular characterization of the *Selinum* spp. [15, 16]. Molecular characterization may further provide database for intensive pharmacological studies in *Selinum*.

It has proven difficult to classify species within the *Selinum* genus because there are no clear morphological synapomorphies and the genus is plagued by synonymies, in which a species is referred to by a different name somewhere else [17]. Several other Apiaceae genera, such as *Angelica* L., *Ferula* L., and *Coriandrum sativum* L., are also treated separately; however, no phylogenetic studies are available in *Selinum*.

Two species of *Selinum*, viz *S. tenuifolium* Wall ex C. B. Clarke (Milk Parsley) and *S. vaginatum* C. B. Clarke (Bhutkeshi), have been identified on the basis of morphology in India. The National Center for Bioinformatics database and UniProt database reveal that studies have only been done on specified regions of the gene or proteins of the study with pharmaceutical or cosmetic motives. However, they do not provide molecular characteristics of *Selinum* spp. [18]. Currently, *rbcL* genes are widely used for phylogenetic analysis within angiosperm families and subclasses, as well as among seed plant groups. However, *matK* evolves 3 times faster than *rbcL* in angiosperms, potentially providing more phylogenetic information and improved phylogenetic resolution among the taxa studied [19, 20]. The present study therefore aims to assess the molecular characteristics of *Selinum* spp. using *matK* and *rbcL* plastid genes in order to derive a phylogenetic relationship within the sp. of *Selinum* as well as with other members of Apiaceae.

## Methods

### Sample collection

The plants were sampled from twelve accessions of *Selinum* collected from different locations representing two states in the Northern Himalayan region of India, namely Himachal Pradesh (HP) and Uttarakhand (UK). Seven *S. vaginatum* C. B. Clarke accessions were collected from various locations, viz SV_1_ — *S. vaginatum*, Chipla kedar, 2691 m (UK); SV_2_ — *S. vaginatum*, Rohtang valley, 2137 m (HP); SV_3_ — *S. vaginatum*, Dhartula, 2378 m (UK); SV_4_ — *S. vaginatum*, Cheena Peak, 1937 m (UK); SV_5_ — *S. vaginatum*, Dhartula, 1819 m (UK); SV_6_ — *S. vaginatum*, Chipla Kedar, 2236 m (UK); and SV_7_ — *S. vaginatum*, Gulaba 2781 m (HP), and five accessions of *S. tenuifolium* Wall ex C. B. Clarke were collected from different locations (ST_1_ — *S. tenuifolium*, Kothi, 2158 m (HP); ST_2_ — *S. tenuifolium*, Rohtang valley, 2350 m (HP); ST_3_ — *S. tenuifolium*, Chopta trek, 2150 m (UK), ST_4_ — *S. tenuifolium*, Chopta trek, 2593 m (UK), ST_5_ — *S. tenuifolium*, Chopta trek, 3178 m (UK)) and stored at −4 °C for molecular studies.

For two target genes, *matK* and *rbcL*, species disparity was accomplished through BLASTn analysis and phylogenetic inference based on maximum likelihood and neighbor-joining method. The disparity index was used to assess the dependability of phylogenetic tree regenerations. The maximum composite likelihood with Kimura 2-parameter model was used to estimate evolutionary divergence between and within all accessions.

### Laboratory reagents, plasticware, and glassware

SIGMA (Sigma-Aldrich, Inc., 3050 Spruce Street, St. Louis, MO, USA) and Gyan Scientific analytical grade reagents and chemicals were used in this study (Lucknow, India). Borosil glassware and Axygen plasticware were used.

### Sterilization of plasticware and glassware

The glassware and plasticwares used for our experiment purpose were washed, cleaned, and sterilized as per the standard protocol recommended for molecular biology researches. All the glassware and plasticware were autoclaved before use, and UV (ultraviolet) irradiation was used as a standard laboratory practice to decontaminate the apparatus. Carryover of DNA from such a previous amplification of the same target almost always results in false-positive amplification. To prevent this, PCR reactions were set up in a separate room than that used for post-PCR manipulations. Autoclaved deionized water (Millipore ZLXS50034, Millipore Bangalore, India) was used for conducting all experiments.

### Genomic DNA extraction

Five *S. tenuifolium* accessions and seven *S. vaginatum* accessions were collected, and their genomic DNA was isolated from young leaves and used as a template for a PCR reaction using a DNA isolation kit (QIAGEN, DNeasy® Plant Handbook). With the respective universal primers, the target DNA regions, namely *matK* and *rbcL*, were amplified.

### Bioinformatics tools

The software used for analyzing the chromatograms of amplicons generated after sequencing by ABI 3130 Genetic Analyzer (Applied Biosystems Inc., USA) was as follows:

- BioEdit program version 3 (Naim & Mahboob. 2020) [21]
- Tamura 3-parameter model (Olmstead & Palmer 1994) [22]
- MEGA version 6.06 (Hartl & Frost 1999) [23]

### Barcoding gene for the study

To evaluate intraspecific and interspecific variations among the accessions of genus *Selinum*, two genes of DNA barcodes (*rbcL*, *matK*) were selected to identify diversity among selected species.

### PCR amplification with matK and rbcL primers

A reaction mixture containing 50 μl of Taq PCR Master Mix to conduct PCR, 22 μl of distilled water, 1 μl of forward primer (10 μM), 1 μl of reverse primer (10 μM), and 1 μl of DNA template (50–80 ng/l) was used to conduct PCR. The PCR conditions are mentioned in Table 1, and primer details are given in Table 2.

**Table 1:** PCR condition

Cycling condition		No. of cycles
Initial denaturation	3 min at 94 °C	
Denaturation	1 min at 94 °C	35 cycles
Annealing	1 min at 50 °C	
Extension	2 min at 72 °C	
Final extension	7 min at 72 °C

**Table 2:** *matK* and *rbcL* primer details

No.	Oligo name	Sequence (5′-3′)	Tm (°C)	GC content
1	*matK* (forward)	CCTATCCATCTGGAAATCTTAG	51	40.9%
2	*matK* (reverse)	GTTCTAGCACAAGAAAGTCG	50	45%
3	*rbcL* (forward)	ATGTCACCACAAACAGAGACTAAAGC	56	42.3%
4	*rbcL* (reverse)	GTAAAATCAAGTCCACCRCG	50	45%

**Fig. 1:**
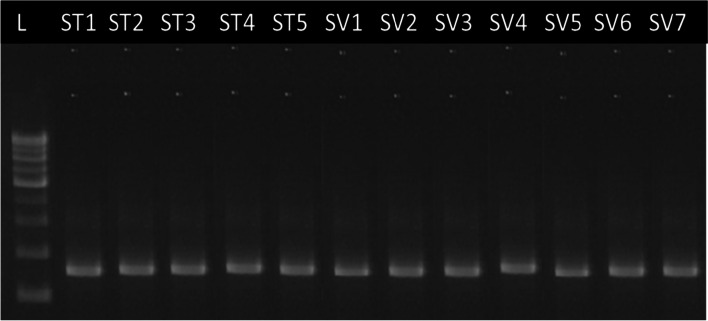
Agarose gel showing amplification of *rbcL* gene

**Fig. 2:**
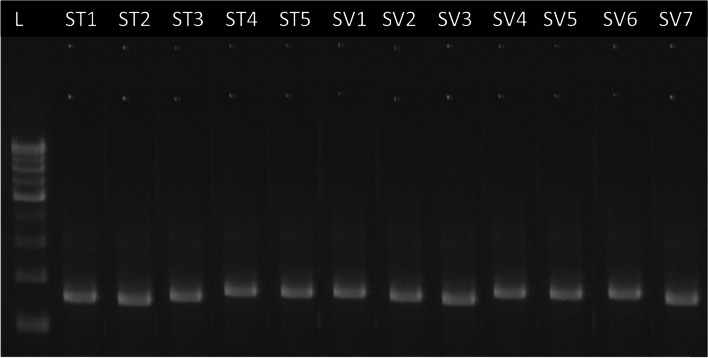
Agarose gel showing amplification of *matK* gene

The PCR products were tested on a 1.5% agarose gel with ethidium bromide in 1X TAE buffer (pH 8.0) to ensure that they were correct. Molecular Imager® GelDocTMXR software was used to examine and archive the gel. With Quantity One software, bands were scored and evaluated (Bio-Rad). By comparing the size of the products to 500 bp DNA Ladder H3 RTU, the size of the products was determined (GeneDirex, cat no. DM003-R500) (Figs. 1 and 2). The nucleotide sequence was converted into an amino acid sequence after the PCR products were verified by electrophoresis, purified, and sequenced using the following comprehensive sequencing procedures (Table 3). The nucleotide and amino acid sequences were aligned using the system software aligner tool to produce a phylogenetic tree based on two statistical data analyses, bootstrapping and pairwise distance, from both nucleotide and amino acid sequences. Table 4 shows the sequencing and features in greater detail.

**Table 3:** Sequencing reaction

**Reagent**	**Reaction volume**	
BigDye Terminator Ready Reaction Mix	4 μl	
Template (100 ng/ul)	1 μl	
Primer (10 pmol/λ)	2 μl	
Milli-Q water	3 μl	
PCR condition for sequencing		
**Step**	**Time and temp**	**Cycles**
Initial denaturation	96 °C for 5 min	**25 cycles**
Denaturation	96 °C for 30 s	
Hybridization	50 °C for 30 s	
Elongation	60 °C for 1.30 min

**Table 4:** Sequencing and chemistry details

S. no.	Instrument/chemical	Detail
1	Sequencing machine	ABI 3130 Genetic Analyzer
2	Chemistry cycle sequencing kit	BigDye Terminator version 3.1
3	Polymer and capillary array	POP_7 pol Capillary Array
4	Analysis protocol	BDTv3-KB-Denovo_v 5.2
5	Data analysis	SeqScape_ v 5.2
6	Software reaction plate	Applied Biosystem MicroAmp Optical 96-Well Reaction Plate

## Results

### Evolutionary and phylogenetic analysis

**Fig. 3:**
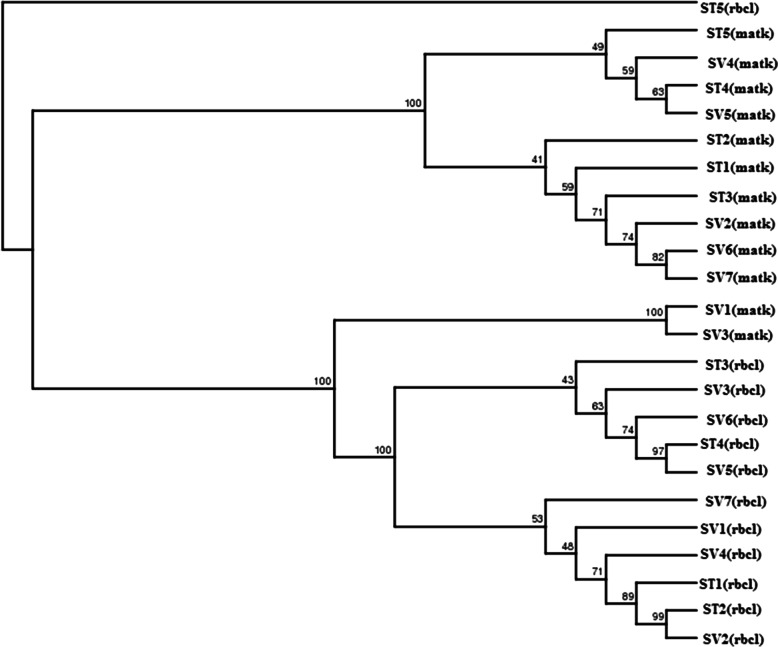
Comparative analysis of 12 different accessions of *Selinum* from *matK* and *rbcL* markers

After DNA sequencing, the twelve accessions of *Selinum* collected from different locations and their molecular sequences of *matK* and *rbcL* markers were evaluated using BLAST method. As a statistical method and a phylogeny test, aligned sequences were used to create phylogenetic trees using the maximum composite likelihood model and bootstrap resampling, respectively. Gene sequences from the twelve *Selinum* accessions were used to create the most likely phylogenetic trees. The numbers at branch nodes are bootstrap values, which show the percentage of bootstrap iterations that support the tree at that specific point of divergence for each phylogenetic tree node. The more the topology of the phylogenetic tree is supported, the higher the bootstrap value. In the phylogenetic tree, species that are adjacent to each other are closely linked. ST5, SV4, ST4, and SV5 have been demonstrated to be closely related (cluster 1) and have a moderate bootstrap support value of 49, whereas ST2, ST1, ST3, SV2, SV6, and SV7 have been grouped together (cluster 2) and have a bootstrap value of 41, showing that this grouping is poorly supported. ST5 is a distant common ancestor of all the accessions. ST3, SV3, SV6, ST4, and SV5 have also been grouped together (cluster 3), while SV7, SV1, SV4, ST1, ST2, and SV2 have been grouped together (cluster 4) based on *rbcL* gene sequences, and both groupings are related to SV1 and SV3, which are clustered separately. Cluster 3 has a moderate bootstrap support rating of 43, whereas cluster 4 has a high bootstrap value of 53, indicating that it is strongly supported. The evolutionary trees of *matK* and *rbcL* are surprisingly similar. ST5, SV4, ST4, and SV5 have been grouped together based on *matK* genes, and all four accessions form a monophyletic group (cluster 1). The phylogenetic tree built using the rbcL genes shows the similar pattern (cluster 4). ST2, ST1, ST3, SV2, SV6, and SV7 have all been demonstrated to be closely related using *matK* genes (cluster 2). Such species’ close association can also be seen in phylogenetic trees based on the *rbcL* genes (cluster 4). ST2 has been shown to be closely associated with ST1, ST3, SV2, SV6, and SV7 (cluster 2) based on *matK* genes, whereas SV7 has been shown to be closely related to SV1, SV4, ST1, ST2, and SV2 (cluster 4) based on *rbcL* genes, as shown in Fig. 3.

### Assessment of genetic diversity in Selinum using matK and rbcL markers

#### Nucleotide variation

**Fig. 4:**
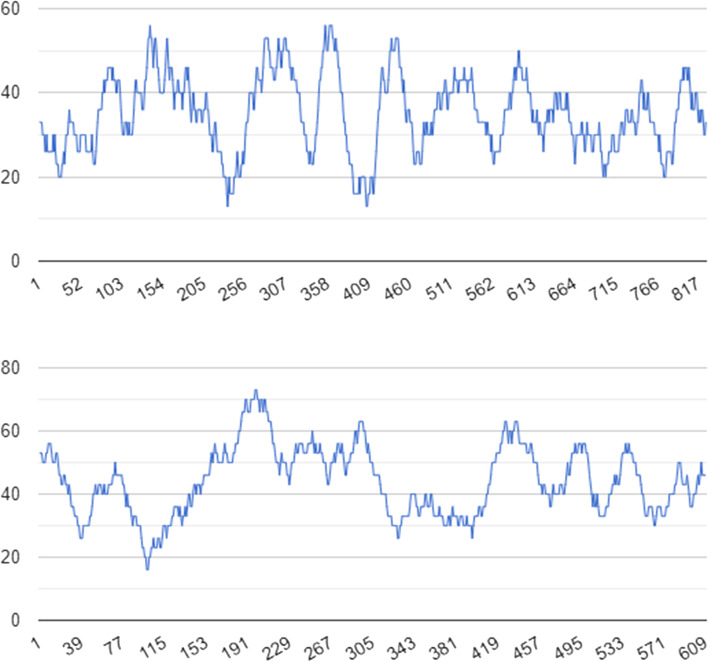
GC content distribution of *matK* (upper panel) and *rbcL* (lower panel) in sequences gene

The length of the *matK* gene sequence ranged from 740 to 855 base pairs (bp), while the *rbcL* gene ranged from 582 to 638 bp. DNA base composition represented as G + C content, where G + C percentage in all species is 40% on average. AT (adenine-thymine) was dominant over GC (guanine-cytosine) in the composition of nucleotides from both genes. In *rbcL*, GC content was 56% and AT was 44%, while in *matK*, GC content was 65% and AT was 35%, respectively (Fig. 4 and Table 5).

**Table 5:** Nucleotide variation in *matK* and *rbcL* chloroplast genes in *Selinum* species

Variable	*matK*	*rbcL*
Length of sequence (bp)	855	638
Number of accessions	12	12
Nucleotide composition	A (36%, 308)	A (27%, 173)
	T (29%, 244)	T (29%, 180)
	G (19%, 163)	G (23%, 150)
	C (16%, 140)	C (21%, 135)

### Phylogenetic relationship between the Selinum species

**Fig. 5:**
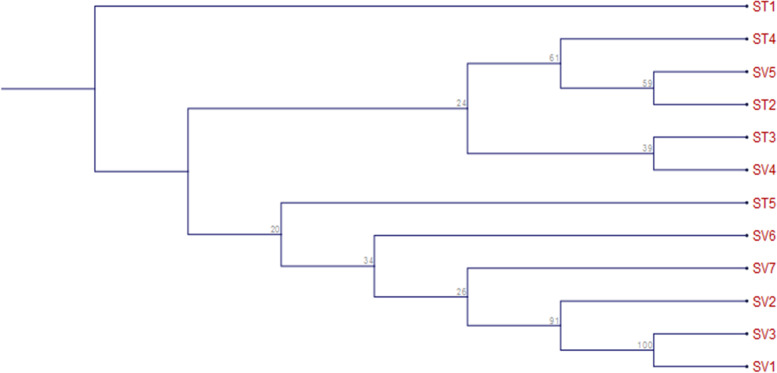
1000 bootstrap replications of phylogenetic tree of partial *matK* gene sequences

Based on partial *rbcL* gene sequences and *matK* gene sequences, the tree construction findings were midpoint rooted. Molecular phylogenetic trees for the *rbcL* and *matK* gene constructed using the maximum likelihood (ML) methods showed approximately identical clustering (Fig. 5). The trees showed that two main divisions of the chosen accessions from different locations of *Selinum* group were clustered together. Four clusters emerged from a cluster analysis of data gathered from the *matK* gene in twelve *Selinum* accessions from various regions. *Angelica sylvestris* was in the first cluster (ST1). The second cluster included *Peucedanum ostruthium* (ST4), *Peucedanum ostruthium* (SV5), and *Angelica sylvestris* (ST2). The third cluster included *Seseli libanotis* (ST3) and *Peucedanum ostruthium* (SV4), while the fourth cluster included *Angelica sylvestris* (ST5), *Seseli libanotis* (SV6), *Seseli libanotis* (SV7), *Angelica sylvestris* (SV2), *Angelica sylvestris* (SV3), and *Peucedanum ostruthium* (SV1) as shown in Fig. 5, and cluster was given in Table 6.

**Table 6:** *Selinum* cluster ID and names for *matK*

Cluster ID	Name
ST1	*Angelica sylvestris*
ST2	*Angelica sylvestris*
ST3	*Seseli libanotis*
ST4	*Peucedanum ostruthium*
ST5	*Angelica sylvestris*
SV1	*Pachypleurum alpinum*
SV2	*Angelica sylvestris*
SV3	*Angelica sylvestris*
SV4	*Peucedanum ostruthium*
SV5	*Peucedanum ostruthium*
SV6	*Seseli libanotis*
SV7	*Seseli libanotis*

**Fig. 6:**
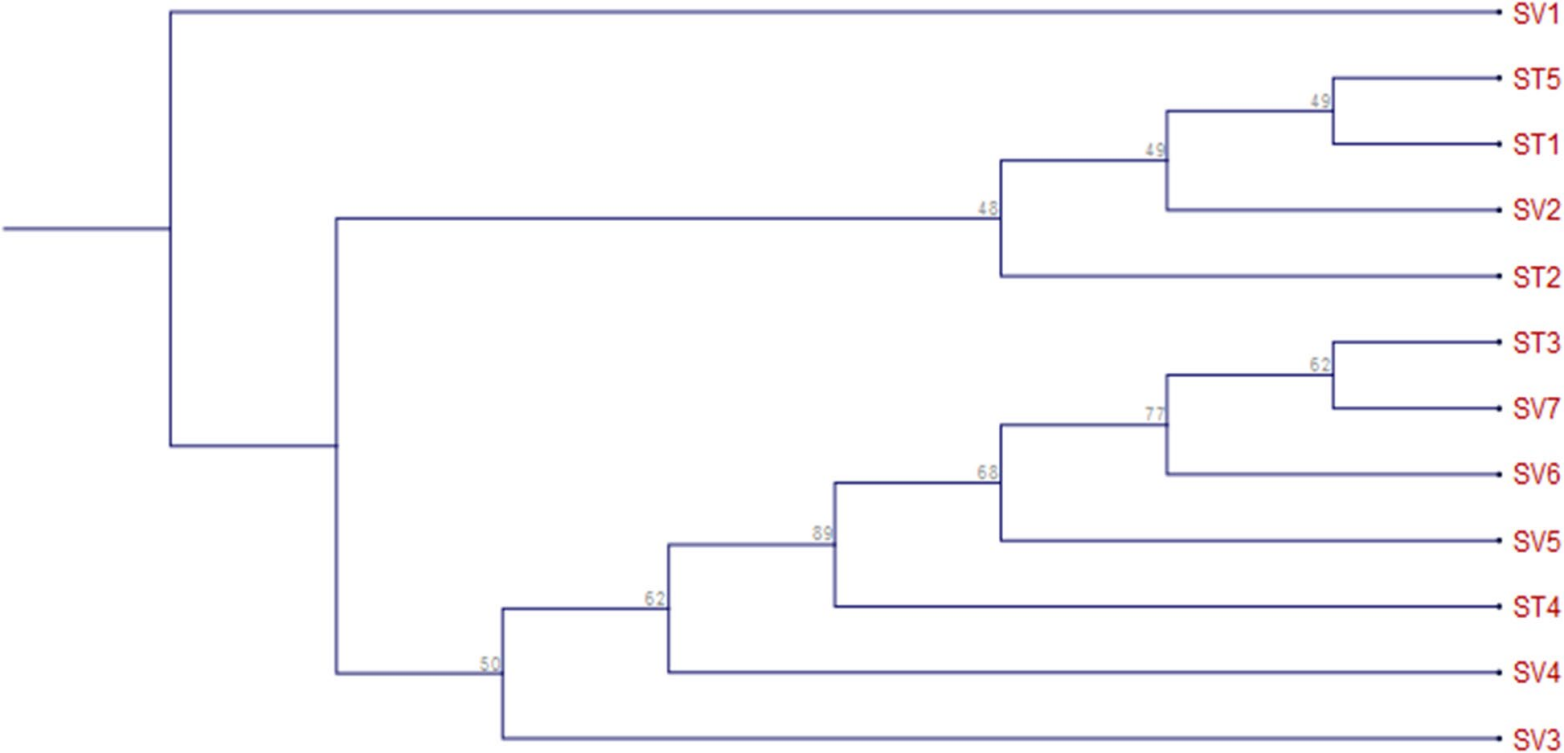
Using 1000 bootstrap replications, phylogenetic tree of partial *rbcL* gene sequences

Clustering analysis from the *rbcL* gene, using *S. vaginatum* as the out-group and *rbcL* phylogenetic trees, found two primary clades and one out-group clade. *Seseli libanotis* is the first clade (SV1). Clade two consist of four accessions: *Peucedanum palustre* (ST5), *Peucedanum palustre* (ST1), *Peucedanum palustre* (SV2), and *Peucedanum palustre* (ST2), while clade 3 consists of seven accessions, *Peucedanum palustre* (ST3), *Peucedanum palustre* (SV7), *Peucedanum palustre* (SV6), *Peucedanum palustre* (SV5), *Peucedanum palustre* (ST4), *Peucedanum palustre* (SV4), and *Peucedanum palustre* (SV3) as shown in Fig. 6 and Table 7.

**Table 7:** *Selinum* cluster ID and names for *rbcL*

Cluster ID	Name
ST1	*Peucedanum palustre*
ST2	*Peucedanum palustre*
ST3	*Peucedanum palustre*
ST4	*Peucedanum palustre*
ST5	*Peucedanum palustre*
SV1	*Seseli libanotis*
SV2	*Peucedanum palustre*
SV3	*Peucedanum palustre*
SV4	*Peucedanum palustre*
SV5	*Peucedanum palustre*
SV6	*Peucedanum palustre*
SV7	*Peucedanum palustre*

### Inter- and intraspecific relationship between matK and rbcL

Based on phylogenetic analysis, twelve accessions, including ST1, ST2, ST3, ST4, ST5, SV1, SV2, SV3, SV4, SV5, SV6, and SV7 from different geographical locations of India, were slightly different from all other accessions and were grouped together (Fig. 3). The samples were also divided into two clades using the maximum likelihood method. Clade 1 was made up of a monophyletic clade that included all of the samples under consideration. The first subgroup was further split into two clades. Clade 1 nested all the *matK* samples under it, while only a single *rbcL* sample was under clade 2. Similarly, group 2 also has two main clusters, both having largely *rbcL* samples.

**Fig. 7:**
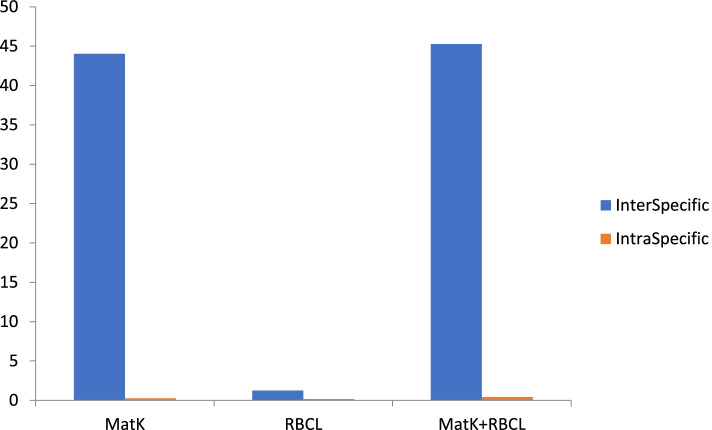
Interspecific and intraspecific relationships between *matK* and *rbcL* gene of twelve accessions of *S. tenuifolium* and *S. vaginatum* collected from different locations

**Fig. 8:**
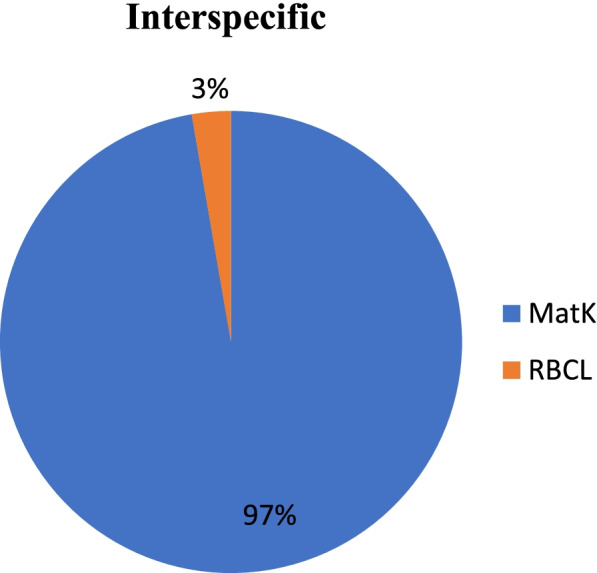
Interspecific relationships between *matK* and *rbcL* gene of twelve accessions of *S. tenuifolium* and *S. vaginatum* collected from different locations

**Fig. 9:**
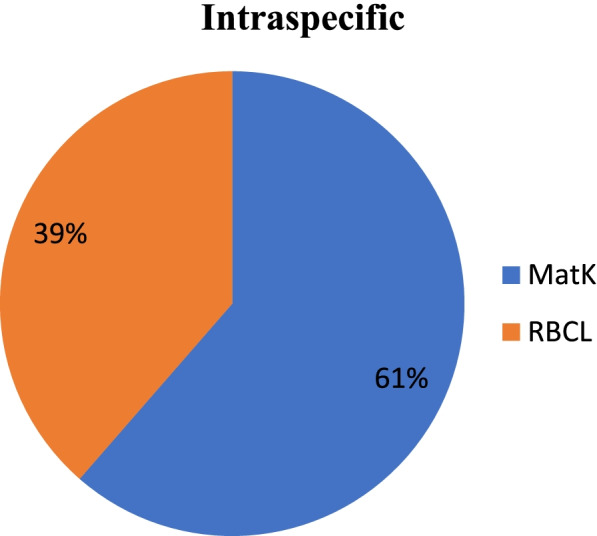
Interspecific relationships between *matK* and *rbcL* and between *matK* and *rbcL* gene of twelve accessions of *S. tenuifolium* and *S. vaginatum* collected from different locations

The intraspecific and interspecific analysis of twelve accessions from different locations of *Selinum* infer that the intraspecific variation between the accessions is very less and interspecific variation as exhibited by *matK* and *rbcL* marker showed variations of different degrees among the species (Fig. 7). *matK* markers showed maximum interspecific variation (97%) and *rbcL* markers only 3% (Fig. 8). Intraspecific variation was also maximum in *matK* (61%) marker fallowed by *rbcL* (39%) (Fig. 9).

## Discussion

The current study found that the DNA identification approach appropriately differentiated all samples up to species level, and that *matK* and *rbcL* are reliable identification markers [21]. It is an accurate method to amplify and sequence, and plastid DNA has played a significant role in building evolutionary relationships and defining limits of species in plants. It is also mostly uniparentally inherited and appears to have little to no recombination, resulting in a small size, structure, and gene order [22]. It is critical to understand the causes of evolution by which processes of genetic polymorphisms across species are converted into genetic divergence between species and genetic diversity by investigating the quantity of nucleotides and patterns of nucleotide variation between and within species. Evolutionary mechanisms, such as selection, recombination, mutation, and population structures, are affected by these diversities [23].

An earlier DNA barcoding research of *Chenopodium murale* produced similar results. According to their findings, the specimen was identified as *C. murale* using BLAST for both the *matK* and *rbcL* genes with 100% sequence matching. Using BOLD, the *rbcL* gene showed a high degree of similarity to various taxa, including *Chenopodium ambrosioides*, *Chenopodium album*, and *Chenopodium ficifolium*, ranging from 96.3 to 100% [24]. The DNA barcode is clearly distinguish between species, with no overlap between intraspecific and interspecific divergence ideally [25]. Furthermore, the effectiveness of any DNA barcoding approach is determined by the degree of intraspecific and interspecific divergence in a single locus or a set of loci [26]. According to the China Plant BOL Group (2011), the combination *rbcL + matK* separates 40% of the sampled species in the matched dataset for the Apiaceae family. Furthermore, among all samples, *rbcL* and *matK* had 32.1% and 38.6% species identification efficiencies, respectively [27]. According to previous research, both *rbcL* and *matK* can be used to identify plants at the species level, because both have a high amplification and sequencing success rate [28]. These two fragments have high identification success rates at the genus and family level [29]. Previous studies reported that genetic distance across accessions could be determined, and that large differences in dissimilarity values could be attributed to genetic differences [30]. For primers *matK* and *rbcL*, for instance, grouping based on genetic distance revealed that one group contains different accessions, while the other contains accessions showing wide variations [31]. Plant DNA barcoding is best accomplished by combining coding and noncoding genetic markers. The highly conserved *rbcL* gene and the more variable *matK* gene are the most widely explored markers in many researches [32, 33].

In this study, all twelve accessions from different locations of *Selinum* were examined, and they all belong to the parsley family, Apiaceae. Five-petalled white flower subgenera were easily distinguished based on the anatomical structure of the leaf. Despite the fact that several morpho-anatomical classification methods for the sections and subsections of subgenus *Selinum* as well as subgenus *Angelica* have been offered, the links between the subsections and their evolutionary processes are still being contested.

India is a rich source of medicinal plants, and there is no data to identify these plants on molecular basis. *Selinum* is a medicinal plant with potential therapeutic value. This study has been conducted to identify *Selinum* using molecular markers because as we go from lower to higher altitude, there are certain changes in morphology and growth within plants which may not be morphologically evident but exist at genetic level. In India, only two species of *Selinum*, viz *S. tenuifolium* Wall ex C.B. Clarke and *S. vaginatum* C. B. Clarke, have been identified on the basis of morphology without any molecular basis ever utilized to authenticate the morphological markers for proper identification of these plants. Various synonyms have been found in *Selinum* and the ambiguity regarding species with various synonyms. *Selinum* species are also hard to distinguish morphologically, because certain species have been given different names by different authors instead of a single genuine name. Therefore, DNA barcoding in this plant would be appropriate tool to establish the correct identification of this species or to differentiate species or genus from other members of this family.

The use of morphological features alone is insufficient to adequately define the genus. When investigating interspecific variations, molecular, anatomical, and biochemical markers are more useful than gross morphological ones. A phylogenetic tree can be formed by comparing the same gene sequence across species within a genus, which can support or provide fresh insights into the present taxonomy and rule out the confusion caused by synonymy in *Selinum*.

## Conclusion

The species boundaries of *S. tenuifolium* and *S. vaginatum* were assessed using phylogenetic tree using twelve accessions obtained from various places in the Indian Himalayas of Himachal Pradesh and Uttarakhand. The sequencing data collected from the samples used during this study together with other samples retrieved from NCBI were analyzed phylogenetically by using maximum likelihood methods resolved essentially identical topologies. The trees that resulted from this method of analysis provided strong evidence of phylogenetic relationships among the samples investigated in the present study. The samples that were being investigated consistently grouped together forming a monophyletic clade with very strong support, suggesting that the investigated samples and *Selinum* evolved from a recent common ancestor of Apiaceae family. This finding implies that the genetic divergence in *Selinum* is phylogenetically informative at the species level. Despite the fact that all of the samples under research were monophyletic, paraphyly was notably noticeable between the samples under investigation and the other *Selinum* samples obtained from NCBI. In reference to the altitude, the genetic diversity maintained a threshold level of only 3% margins encoded by the comparative analysis of *matK* and *rbcL*.

## Data Availability

The datasets used and/or analyzed during the current study are available from the corresponding author on reasonable request.

## Abbreviations

matK: Maturase K
rbcL: Ribulose 1,5-bisphosphate carboxylase
PCR: Polymerase chain reaction
DNA: Deoxyribonucleic acid
BLAST: Basic local alignment search tools
AT: Adenine-thymine
GC: Guanine-cytosine
ML: Maximum likelihood
